# Stress reduction from landscape painting and live nature viewing: a comparative experimental study

**DOI:** 10.7189/jogh.15.04146

**Published:** 2025-05-16

**Authors:** Linda Powers Tomasso, Piotr Białowolski, John D Spengler

**Affiliations:** 1 Department of Environmental Health, Harvard T.H. Chan School of Public Health, Boston, Massachusetts, USA; 2 Department of Economics, Kozminski University, Warsaw, Poland; 3 Human Flourishing Program, Institute for Quantitative Social Science, Harvard University, Cambridge, Massachusetts, USA

## Abstract

**Background:**

Societal trends indicate a decreasing frequency of visits to natural settings and an increasing withdrawal from such environments. Separation from natural environments may lead individuals to miss out on the health benefits associated with direct exposure to nature, including stress reduction and positive mood enhancement. Given these trends, surrogate exposures to live nature exposure might comparably reduce stress and are worth exploring. This experimental study contrasts stress reduction from viewing landscape paintings *vs*. live nature in situ.

**Methods:**

We used sensor-monitored skin conductance and survey instruments on 37 older adults who are regular museum visitors to measure individual stress recovery and improvements in mood indicators for each nature viewing treatment, outdoor park and indoor landscape paintings, conducted during a museum-based educational programme in May 2022 in the Northeastern USA.

**Results:**

Difference-in-difference analyses on survey data identified stress reduction of comparable statistical magnitude following both viewing types. However, significantly lower average levels of physiological arousal as measured by skin conductance were observed among park viewers (α = 0.126 for park viewing *vs*. α = 1.172 for gallery viewing). Regression analysis comparing slopes and rates of change in stress reduction during the calming events revealed a faster rate of stress reduction during the gallery viewing (β = −0.217 for the gallery *vs*. β = −0.066 for the park).

**Conclusions:**

Findings from this research could be relevant for populations without live nature access or disinclined to go outdoors by offering alternatives to first-person nature contact that reduce stress and enhance positive affect as observed following nature viewing.

Westward US expansion invited many premier mid-nineteenth century painters to capture majestic landscape vistas unknown back East. The artistry of Thomas Moran, Albert Bierstadt, and Jasper Cropsey transported the Rockies and Sierra Nevada to eastern museums, where residents of industrialising cities discovered nature imagery on a grand scale. Paintings proxied these landscapes with such realism that Congress was persuaded to protect these vistas through the Origin Act of 1916 creating the US National Park System (NPS). Soon after, early photographers like William Henry Jackson working in the new medium of stereoscopic cameras introduced western mountain passages and monuments to wider audiences [1]. Carleton Watkins’ photos of Half Dome were instrumental in convincing politicians to safeguard Yosemite Valley through NPS designation [2,3]. A half century later, Ansel Adams’s dramatic photos of unyielding Western landscapes led to a US Department of the Interior commission to capture the NPS in print. On walls, screens, or gel prints, nature imagery fascinates and soothes. The desire to deliver therapeutic nature exposure draws on evidence that associates nature contact with positive emotions [4], anxiety reduction [5–7], lower rumination [8,9], and likelihood of dementia [10]. But to what degree can a facsimile stand up to live nature in delivering nature contact’s key health benefits: stress reduction and positive mood enhancement?

EO Wilson’s Biophilia theory posits that the human species harbours an innate connection to nature and other forms of life [11–14]. Scientific research provides ample psycho-physiological evidence for influence of biophilic response in natured *vs*. built environmental settings [15–18]. Individuals now spend on average 90–95% of their time indoors [19,20]. Life indoors limits time in nature, a traditional antidote to stress and attentional fatigue. Mental stress is the psychiatric basis found to trigger cardiovascular disease response [21], overload of the capacity for normal allostatic response [22], and cell damage, pro-inflammatory response, and compromised DNA repair mechanisms [23]. The digital era further has been shown to compound stress accumulation, compromising physiological and psychological health through social media-induced mental health problems [24–26]. Yet where screen time predicted higher anxiety, stress, and depression, time in nature was a protective factor [27].

Stress reduction ranks among the better documented outcomes of nature contact. A primary theoretical framework to have emerged from empirical research on the physiological and psychological benefits of nature contact is the Stress Reduction Theory [28–31]. It posits that humans’ affective reaction positively ties to underlying autonomic nervous system responses grounded in evolutionary biophilia. Researchers have expounded on this foundational work, adding compelling evidence supporting nature’s ability to quiesce stress and anxiety [29,32–35]. The biological mechanisms through which cognitive and affective benefits of nature exposure reduce stress are complex and likely to occur in part as conditioned behavioural and psychophysiological responses [36–38]. Stress reduction through time spent in nature was heavily examined under the COVID-19 pandemic. Subjective data associated greater access to neighbourhood greenspace with lower perceived stress among individuals sheltering-at-home [39–41].

Museums have their own investigative focus as sites of social interventions intended to maintain health and well-being, especially for older populations [42]. Kaplan found museums to be inherently restorative environments owing to factors of being away, spatial extent and coherence, and interest and engagement [43]. Studies have considered the overall psychological outcomes of viewing paintings [44–46]; Fekete [45] contextualised art viewing within an array of aesthetic experiences as a direct positive impact on autonomic nerve response but did not give specific attention to landscape painting. Moreover, pictorialised nature in two- and three-dimensional forms has occupied a central role in studying of nature-induced stress responses (**Box 1**).

Box 1 **The use of nature surrogates in researching stress reduction**

The use of pictorial nature has characterised research on nature-induced stress reduction since its origins [47,48]. Visual surrogates to nature exposure on human emotional and cognitive experience have included photos [6,9,47,49–51], documentary videos [52–54], and more recently virtual reality as indirect opportunities to experience nature [55–58]. Visual features of naturalness encompass restorative components which relate to cognitive functioning and restoration of executive functioning used outdoor images [59,60]. A study examining how nature exposure modifies neurological response to stress placed landscape photos as stimuli within functional magnetic resonance imaging chambers [61]. Some studies have incorporated outdoor viewing as the true analogue of interest to simulated nature. The literature review generally favours live outdoor nature for evocation of greater health benefits. Jo et al.’s [62] systematic review of studies of indoor nature experiences concluded that viewers of real natural stimuli exhibited more relaxed body responses and positive cerebral and autonomic nervous system response when compared to display stimuli, *e.g*. 3D images, virtual reality (VR) and natural landscape videos. The advancement of visual technologies like VR is reflected in experimental designs comparing live *vs*. synthetic nature [63,64]. Investigators using VR technology to compare human response toward live nature *vs*. virtual nature scenes found that virtual representations of nature elicited psychological reactions similar to indoor biophilic environments [65–69].

Acknowledging these precedents, the research presented here revisits an earlier practice of surrogate nature viewing – landscape painting – to compare its effectiveness against current evidence of stress reduction experienced outdoors. The study design takes advantage of the unique conjunction of Hudson River School landscape paintings exhibited in a museum overlooking an Olmsted-designed urban park, thus allowing us to compare the effects of stress reduction measured in study participants through in situ nature viewing in both live and simulated form. Use of real-time wearable bio-monitoring sensors and standard psychological self-assessments gauged participants’ physiological and psychological responses to these two nature exposures, thereby providing evidence to test our hypothesis.

## METHODS

### Study site

**Figure 1:**
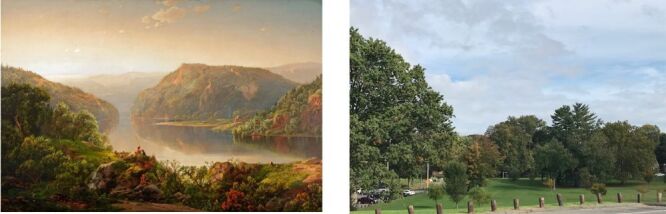
**Panel A.** View in Vermont by William Louis Sonntag, c. 1874, Oil on canvas, permanent collection of the New Britain Museum of American Art, Judd Landers Fund. **Panel B** View of Olmsted-designed Walnut Hill Park, New Britain, CT.

The New Britain Museum of American Art, founded in 1903 as the country’s first Museum dedicated to the collection of American art, has a superlative collection of nineteenth century landscape paintings in its permanent collection (**Figure 1**, Panel A). The Museum overlooks Walnut Hill Park designed by famed landscape architect Frederick Law Olmsted in 1865 (**Figure 1**, Panel B). Weather conditions on study day were sunny with an outdoor temperature of 70°F (F) and sunny; indoor gallery temperature remained at +70 ± 5°F, with relative humidity kept at 50 ± 5% as per national collections management policy.

### Study population

Study participants were recruited through the host Museum’s educational programming web portal which advertised a scientific study inviting audience engagement. Prospective enrolees were able to read in advance the eligibility criteria for research participation and self-select into the study by pre-registering attendance on the Museum event website. Eighty individuals pre-registered, and 40 attended the presentation. Thirty-seven individuals gave oral consent to wear biometric sensors to monitor their galvanic skin response (GSR) levels. Consenting study participants completed a baseline survey containing questions on age, gender, race, urban density in childhood, self-reported physical and mental health, caffeine intake, feeling rested prior to experiment, and current stress level. Nature affinity, frequency in outdoor nature, and prior negative and positive experiences in nature were also recorded.

### Study design

This study was conducted in the form of a public lecture, ‘Nature Viewing as Science,’ presented by the first author in conjunction with the celebration the Olmsted Bicentennial. A randomised crossover study design was employed for which each participant served as his/her own control to minimise between-subject variation. Order of treatment was randomly assigned as participants entered the lecture hall and completed study enrolment (Figure S1 in the **Online Supplementary Document**). No identifiable information was collected on any individuals, as participants were assigned a random, non-identifiable ID number for the experiment. The study was conducted according to the guidelines of the Declaration of Helsinki and approved by the Institutional Review Board of Harvard T.H. Chan School of Public Health (protocol code IRB22-0382 on 29 April 2022). Study participants received no compensation for their time.

### Study tools and outcome measures

Participants’ physiological and psychological responses to stress-related stimuli were gathered through wearable bio-monitoring sensors and standard validated psychological indices. NeuroLynQ biometric sensors [70] were used to measure real-time skin conductance levels indicating physiological stress. Two electrodes placed on the index and middle fingers of consenting participants and sensors affixed to the wrist of each participant’s non-dominant hand measured individual electrodermal activity response. These electrodes streamed real-time data to an on-site desktop computer. Sensor output captured emotional arousal expressed as galvanic skin response (GSR) through continuous dermal readings of raw GSR signal in micro siemens on each individual streamed at a sampling rate of five hertz. Data collected directly to the secure digital card on the sensor was recorded at 256 Hz [71]. Participant transitions between Museum viewing areas eliminated the use of heart rate variability as a second physiological measure due to potential confounding with normal physical activity and different physical baselines of heart health.

Each treatment group, park *vs*. gallery nature viewing, was additionally monitored by a separate Shimmer NeuroLynQ system to provide real-time analysis of group-level response rates to the viewing stimuli. Real-time analytics were based on the percentage of the audience having a measurable reaction, *i.e*. response rate, at any given time. Response rates were calculated by analysing the GSR of each participant and categorising their response as none, some, or high level. Individual participant results were then aggregated by calculating the percent of audience in each response category at each moment.

In addition to the physiological data, participants also completed immediately following each viewing treatment the six-item short-form State-Trait Anxiety Inventory (STAI-6) [72,73] and the 37-item short-form Profile of Mood States Questionnaire (POMS) [74,75] to self-assess subjective mood states and transient anxiety level. The short form STAI-6 consists of six anxiety-positive questions and six anxiety-negative questions intended to differentiate more permanent personality features (trait) from those representing transitory emotional response (state). The STAI-6 is a four-level scale of ascending anxiety, reversed for anxiety-negative items, and has demonstrated high correlation with the full 20-item form, with internal reliability exceeding 0.90 [72].

The short-form POMS Questionnaire describes seven emotional dimensions: six negative transient mood states: depression, vigour, confusion, tension, anger, and fatigue; and one positive state, vigour. Each mood is further parsed through subset descriptors scored 1–5, which are then compositely tallied by domain. This widely used, abbreviated POMS yields results highly comparable to estimates from the longer 65-item version (*P* > 0.95) [34,76]. Finally, each participant is assigned a total mood disturbance (TMD) score [77] from the short-form POMS; higher TMD scores indicate a greater degree of mood disturbance:

TMD = Depression + Tension + Anger + Fatigue + Confusion − Vigour

### Experimental procedures

The study took place and all data collected on 1 May 2022. After consenting participants were seated in the lecture hall and randomly assigned to treatment order, a NeuroLynQ staff member demonstrated how to wear the biosensors so that study participants themselves could affix the sensors to their wrists. Attendees were reminded of their right to terminate their study participation at any point during the process. The preparation process took about ten minutes, during which participants settled into a calm state for baseline physiological data capture and baseline data survey completion.

**Figure 2:**
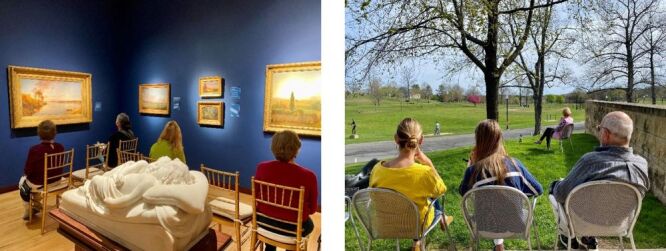
**Panel A.** Actual nature viewing conditions, indoor gallery viewing of landscape paintings. **Panel B.** Outdoor viewing of Olmsted-designed Walnut Hill Park.

A 25-minute presentation discussing landscape painting composition and biophilic stress reduction preceded the scientific portion of the study. All audience members then watched a five-minute video montage of an emergency vehicle stalled in New York City traffic intended to induce a moderate level of stress over baseline conditions. After the video concluded, participants transitioned to one of two nature settings adjacent to the auditorium for an uninterrupted seven-minute viewing session. A seven-minute duration was selected based on precedent in cognitive studies involving nature and music exposure [78,79]. Group 1 proceeded to the exhibition gallery hosting The New Britain Museum of American Art’s permanent collection of landscape paintings just outside the lecture hall and was seated in a chair facing the landscape paintings. Group 2 concurrently exited to the Museum’s outdoor terrace adjacent to the lecture hall where chairs were set up facing the park. In both nature viewing areas, seats were equidistantly spaced; gallery chairs set at five feet in front of the artwork so that all participants had full and unobstructed view of one or more framed landscape painting (**Figure 2**, Panels A–B). Both treatment groups silently observed their respective nature form while individual stress levels were monitored continuously. Following this first treatment, both groups returned to the lecture hall for a two-minute reset. They then viewed a second five-minute video montage of different traffic scenes. Treatment groups then switched order, with Group 2 exiting to view the landscape exhibition, and Group 1 retreating outside to view the live nature. At the end of seven-minutes, both groups returned to the lecture hall to debrief.

Physiological and psychological responses were measured repeatedly during the experiment capturing discordant (traffic) and concordant (nature) sensory input. Real time GSR data was continuously recorded, and post-experience POMS and STAI tests were administered in situ after each treatment for 37 individuals. The same procedure was repeated under both traffic and nature viewing conditions. The four completed psychological profiles per participant yielded a total of 5940 subjective data points. Before and after sensor readings taken on n = 32 individuals yielded 123 observations per outcome measure, or 492 total on each individual. The formal study concluded with devices removed from participants, and a brief discussion session was conducted during which real-time biomonitoring results were shared with participants.

### Statistical analysis

Standardisation of data was performed on the sensor readings taken on each respondent to reduce the differences in magnitude of observed skin conductivity levels between individuals. The procedure was justified because respondents experienced different average readings over the course of the experiment which could potentially introduce bias into the results. Due to this standardisation, the results might be treated as relative to the mean level of stress observed during the whole study period and also be comparable between respondents. Consequently, each respondent contributed equally to the observed average differences across sensor readings. Readings across respondents were compared to identified experiment stages associated with highest and lowest levels of stress on average. Sensor readings were also utilised in regressions to track responses to different exposures over the duration of each experiment section.

Two-sample *t* tests with equal variances were performed between the first traffic exposure and the final nature viewing for subjective psychological responses assembled by mood domain. Participants’ random ID was treated as a random intercept to control for confounding by individual characteristics. Each treatment scenario was separately examined to obtain beta estimate and 95% confidence interval (CI) for each outcome measure, and an alpha level of 0.05 was used to determine statistical significance.

To evaluate the differences in stress and anxiety reduction between participants exposed to park viewing and those exposed to gallery painting viewing, we employed a difference-in-differences (DiD) approach. This method allowed us to assess changes in self-reported stress and anxiety levels, measured using the STAI and the POMS scales, as well as the individual components of both scales.

The study began with participants being exposed to a traffic film designed to induce agitation, which served as the baseline measurement. Following this, participants were exposed to either park viewing or gallery painting viewing. Stress and anxiety levels were measured both after the traffic film and after the subsequent exposure. To compare the effects of gallery painting viewing with those of park viewing, the treatment was defined as exposure to gallery painting viewing, while the control group was defined as exposure to park viewing. Each participant experienced both conditions, either starting in the control group and then moving to the treatment group, or vice versa. The DiD approach estimated the interaction effect between the treatment group (park *vs*. gallery) and the measurement occasion (post-traffic film *vs*. post-exposure). This interaction term captures the differential impact of park viewing *vs*. gallery painting viewing on stress and anxiety reduction.

To account for potential confounding factors, we included several control variables in the analysis. These variables used to model affect change were age (≤60, reference category), sex (female), childhood urban density (suburb, with four levels being large city (≥250 000 population); suburb; small town (≤15 000), rural area), overall stress level prior to the experiment (1–5 scale), and nature affinity (level 1, 1–7 scale). By including these controls, we aimed to isolate the effect of the treatment (park *vs*. gallery exposure) on stress and anxiety reduction, while accounting for individual characteristics and baseline stress levels.

The DiD approach was applied to each outcome variable, allowing us to assess whether the change in stress and anxiety levels differed between the two groups after exposure to the respective conditions. This approach provided a robust framework for evaluating the differential effects of park and gallery exposure on stress and anxiety reduction.

All analyses were performed using Stata, version 17 (College Station, Texas, USA).

## RESULTS

### Descriptive statistics

Descriptive statistics are presented on the 37 participants (Table S1 in the **Online Supplementary Document**). Participants were on average 68.51 years old (standard deviation (SD) ± 13.61); two-thirds were female and one-third male; all identified as white race. Good, very good or excellent physical health characterised 97.3% of participants and 100% self-reported equivalent mental health ratings; 86.5% reported good sleep quality the previous night; 43.2% had a caffeinated drink within six hours prior to the experiment. The mean self-reported stress level on a 1–5 (low-to-high) scale was 2.68 (SD ± 1.06). Nature affinity was self-assessed through the single-item measure Inclusion of Nature in Self [80], with participants reporting a mean nature affinity score of 5.03 (SD ± 1.25). Several covariables of interest collected in the baseline survey were dropped from analysis due to the homogeneous nature of the study population, including current urban density, previous experience in nature (95% having had a positive experience), and caffeine consumption, all of which proved non-significant in univariable regression testing.

### Summary findings

Data generated by the NeuroLynQ sensor monitoring allowed for some overall observations to be drawn. First, the order effects in the two exposures were negligible, suggesting that the physiological experience of both viewing groups appear quite similar. Surprisingly, the GSR response rates was only higher for the second traffic video than either the park or gallery nature viewing despite its piercing soundtrack; the first traffic video elicited below average responses. Second, in both the park and the gallery environments, participant response rates were starting from initially relatively high levels as they were settling in, and then dropped, suggesting both scenarios were relaxing in themselves. Third, viewing nature in park and paintings elicited different GSR response patterns. Park viewers experienced an initially lower rate of physiological arousal than the gallery viewers and a stable rate of relaxation. Fourth, viewers of paintings had lower response rates after the initial settling-in period, signalling deeper relaxation across the seven-minute viewing window.

### Effect of stressor and nature treatments on physiological measures

Different stress levels between participants during the experiment were demonstrated by their skin conductivity measures (**Table 1**; Figure S2 in the **Online Supplementary Document**). During the first traffic exposure, the average stress level paradoxically measured below the mean GSR readings across the four exposures. Moreover, this average reading was not significantly higher or lower with respect to the first nature exposure (gallery or park depending on the group). Only during the second exposure to traffic did participants experience significantly higher stress. Stress levels measured 0.317 on standardised a scale during that phase of the experiment, and it was the only part of the experiment when the readings registered above average. Sensor measures for the second traffic exposure were also significantly higher than at any other time during the experiment, including those occurring prior to (first traffic and first nature exposure) as well as subsequently (second nature exposure). The second nature exposure was also associated with readings of skin conductivity that were below average (−0.098 combined average for gallery or park on the standardised scale). When comparing skin conductivity readings between the gallery and park settings – each of which could have been presented either as Exposure 2 or Exposure 4 – we observed that skin conductivity levels were significantly lower for the park compared to the gallery (−0.234 *vs*. 0.000). Additionally, we examined whether the order of exposure (*i.e*. whether the gallery or park was the first or second nature exposure for a given respondent) influenced skin conductivity levels. The differences in skin conductivity levels for either the park or gallery were not statistically significant between participants who experienced the given exposure as their first nature exposure (Exposure 2) *vs*. those who experienced it as their second nature exposure (Exposure 4). For the park, the *P*-value of the *t* test was 0.052, while for the gallery, it was 0.984.

**Table 1:** Average levels of standardised measures of skin conductivity collected from participants during different phases of the experiment

Measure	Exposure	Exposure 1: traffic	Exposure 2: gallery or park	Exposure 3: traffic	Exposure 4: gallery or park	Gallery (either exposure)	Park (either exposure)
Average*		−0.043	−0.119	0.317	−0.098	−0.000	−0.234
Statistical significance of the difference (*P*-value)	E1: traffic		0.289	0.000	0.418		
	E2: nature	0.289		0.000	0.689		
	E3: traffic	0.000	0.000		0.000		
	E4: nature	0.418	0.689	0.000			
	Gallery						0.000
	Park					0.000	

*The results for each respondent were standardised over the course of the experiment so that each participant’s reading had an average of zero and standard deviation of one during the course of the experiment. The first exposure measurement corresponds to gallery for Group A and park for Group B and the opposite for the second exposure measurement.

The second part of the experiment concerns the effects observed during each exposure phase. It was assumed that there would be a time effect of being exposed to either traffic, gallery or park during the course of that exposure. We used the following model to evaluate the time effects of being exposed to traffic gallery or park:

*skin_conduc_t,i_ = α + βt + γt^2^ + ε_t,i_*

This equation assumes that the de-stressing effects of nature increase or decrease linearly at the rate β but that there is an additional effect that either accelerates or decelerates this growth and is associated with γ, that is the quadratic term in the regression (**Table 2**).

**Table 2:** Regression estimates indicating the levels of skin conductivity over time during specific exposures

Measure	Exposure	Exposure 1: traffic	Exposure 2: gallery or park	Exposure 3: traffic	Exposure 4: gallery or park	Gallery (either exposure)	Park (either exposure)	
Estimated coefficients (standard errors)	α	1.188* (0.108)	0.781* (0.079)	1.362* (0.054)	0.607* (0.053)	1.172* (0.065)		0.126† (0.061)	
	β	−0.276* (0.029)	−0.178* (0.019)	−0.213* (0.019)	−0.119* (0.013)	−0.217* (0.016)		−0.066* (0.015)	
	γ	0.009* (0.002)	0.006* (0.001)	0.006* (0.001)	0.003* (0.001)	0.006* (0.001)		0.002‡ (0.001)	
Model fit	R^2^	0.21	0.16	0.39	0.14	0.27		0.04	
	Number of observations	432	528	432	572	594		506	
	Time when skin conductivity stabilises *t* = −β/2γ§	15.4	15.8	18.0	18.8	16.9		17.0	

*P < 0.001

†*P* < 0.05

‡*P* < 0.01

§t is measured in 20-s intervals, which is *t* = 0 corresponds to the beginning of particular exposure, *t* = 15 corresponds to the end of traffic exposure which lasts for five minutes, and *t* = 21 corresponds to the end of gallery/park exposure which lasts for seven minutes.

In all cases, both for stressful traffic exposures and calming nature exposures, skin conductivity readings followed a U-shaped path. Initial readings were relatively high indicating a certain level of agitation at the beginning of each exposure. Gallery viewers began their exposure at a higher alpha, displaying an initial arousal point that may reflect higher residual stress owing to a shorter walk to the gallery than to the park and the need to orient oneself within the landscape gallery to a single painting selection for viewing. This effect contrasted with a much faster GSR decline indicated by β = −0.217 *vs*. −0.066 for the park.

After three minutes exposure time, gallery viewers reached a deeper level of relaxation. Subsequent readings were lower within each exposure window. The first traffic exposure showed the fastest observed decline, *i.e*. the largest beta, and the park exposures the slowest. The park-exposed group also started from the lowest level of initial agitation, *i.e*. the lowest alpha, as measured by skin conductivity levels. Over the course of each exposure, the level of agitation stabilised. This stabilisation point occurred approximately five minutes after the beginning of the first traffic exposure and about six and half minutes (18.8 20-second intervals) for the second nature exposure.

### Effect of stressor and nature treatments on psychological measures

The STAI and POMS inventories captured psychological measures after each exposure through survey-taking. After viewing the two traffic videos, respondents reported high levels of tension (Tense = 2.868, close to average of ‘moderately so’), fatigue (Fatigued = 2.412) and anger (Angry = 2.308). Of the six mood changes addressed by the POMS survey, only eight of the 37 questions relating to ‘anger,’ ‘fatigued,’ or ‘confused’ registered *P*-values of significance or marginal significance to different viewing conditions following the first traffic exposure. The differences in the remaining 29 mood questions recorded ‘before’ nature viewing and all 37 mood items ‘after’ the nature viewing interventions proved non-significant.

**Figure 3:**
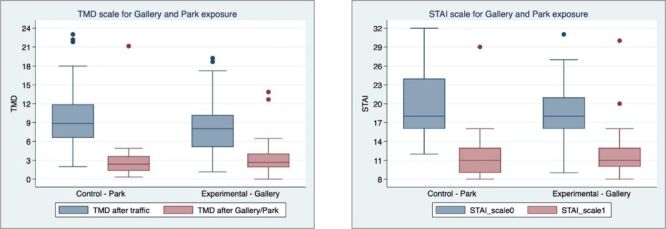
Mean differences in Total Mood Disorder scores (**Panel A**) and State-Trait Anxiety Inventory Scale scores (**Panel B**) during the 5-minute viewing window of each environment in four post-viewing groups. No significant difference resulted from effect of treatment. STAI – State-Trait Anxiety Inventory, TMD – total mood disturbance.

Stress-reduction following exposure to landscape paintings and park immersion was observable and highly significant for all measures of self-reported STAI and POMS as confirmed by highly significant *t* test values for each of the eight mood dimensions in STAI and six dimensions in POMS (**Table 3**). The composite measures for the two instruments provided very different readings following the traffic and after the calming event. Despite strong calming effect observed following painting or nature viewing, there is no evidence that either intervention approach (landscape painting or nature park viewing) differed in its psychological effect on stress mitigation. This outcome was confirmed by the difference-in-differences approach which was not statistically significant for any measure of STAI or POMS as well as for the composite measures provided by the instruments **(****Figure 3**, Panels A–B).

**Table 3:** T-test scores indicating average readings and difference-in-difference estimates for nature viewing treatments*

	State-Trait Anxiety Inventory (STAI)†								
	**Calming effect of paintings/park**			**Difference in differences**					
	**Traffic**	**Paintings/park**	**T-test (95% CI)‡**		**Traffic (treatment)**	**Traffic (control)**	**Paintings (treatment)**	**Park (control)**	**DID (95% CI)‡**
Tense	2.868	1.309	1.559§ (1.30, 1.82)		3.247	3.311	1.665	1.847	0.256 (−0.32, 0.83)
Depressed	1.779	1.103	0.676§ (0.42, 0.94)		2.044	2.257	1.511	1.601	0.123 (−0.42, 0.67)
Angry	2.308	1.103	1.206§ (0.93, 1.49)		3.205	3.424	2.138	2.112	0.246 (−0.37, 0.89)
Clear-headed	2.662	3.294	−0.632§ (−0.96, −0.31)		2.830	2.630	3.430	3.380	−0.150 (−0.82, 0.52)
Vigorous	2.338	2.676	−0.338¶ (−0.64, −0.04)		2.277	2.217	2.411	2.779	−0.429 (−1.02, 0.16)
Fatigued	2.412	1.485	0.926§ (0.62, 1.24)		3.453	3.567	2.487	2.692	−0.092 (−0.74, 0.55)
Confused	1.897	1.221	0.676§ (0.40, 0.95)		2.818	3.190	2.351	2.283	0.440 (−0.12, 1.00)
Good-natured	2.250	3.353	−1.103§ (−1.41, −0.79)		1.643	1.453	2.743	2.515	0.037 (−0.62, 0.70)
Composite measure‡	19.015	11.897	7.118§ (5.58, 8.66)		23.016	24.449	16.750	16.667	1.515 (−1.78, 4.81)
	**The Profile Of Mood States (POMS)†**								
Tense	1.939	0.309	1.630§ (1.32, 1.94)		2.676	2.698	1.148	1.047	0.123 (−0.56, 0.81)
Depressed	0.950	0.167	0.783§ (0.45, 1.11)		1.944	2.027	1.307	1.312	0.077 (−0.58, 0.74)
Angry	1.379	0.145	1.234§ (0.94, 1.53)		2.091	2.407	1.099	1.090	0.324 (−0.31, 0.96)
Vigorous	1.331	1.909	−0.578¶ (−0.93, −0.21)		1.226	0.948	1.618	1.703	−0.363 (−1.01, 0.29)
Fatigued	1.373	0.309	1.064§ (0.74, 1.39)		1.676	2.194	0.834	0.875	0.477 (−0.20, 1.16)
Confused	0.979	0.218	0.761§ (0.45, 1.07)		1.626	1.925	0.996	0.944	0.351 (−0.28, 0.99)
Composite measure‡	9.235	3.238	5.996§ (4.56, 7.44)		12.533	14.182	7.640	7.444	1.845 (−1.22, 4.91)

CI – confidence interval, DID – difference in differences

*T-test scores for all eight scale items on the State-Trait Anxiety Inventory and the six items on the Profile of Mood States indicate response estimates between viewing live (park) and landscape paintings following traffic video did not significantly differ. Composite measures for both scales showed significant difference in difference value (*P* < 0.01 level).

**†**Self-assessed measures use different scales. The State-Trait Affect Index (STAI) uses a four-point scale of negative to positive value, while the Profile of Moods States (POMS) employs a five-point scale of increasing value. Scales were treated as continuous for *t* tests and DIDs.

‡95% CI for *t* test and 95% bootstrapped CI for DiD estimates. Composite measures represent the sum of eight mood subdomains for each scale. *t* test measures difference in mean response between exposure to Traffic *vs*. Nature (either painting or park); treatment is paintings, control is park. Difference in differences indicates stress reduction effect (role stress reduction from viewing landscape painting *vs*. live nature in park); difference-in-differences estimator with covariates was used.

§*P* < 0.01.

¶*P* < 0.05.

## DISCUSSION

The phenomenon of nature seeking is millennia-old, and artistic imitation of nature has extended this tradition. Contact with visual nature surrogates have long filled the gap of first-person nature contact. Nature as the object of mimesis was articulated by Greek philosophers, and biophilic references infused Chinese calligraphic paintings, Persian ornamentation, and the church portals of Medieval Christendom. The 19th century witnessed a shift toward nature from aesthetic pleasure to biophilic need with the onset of industrial change. Transcendentalist writings and paintings romanticised nature as a soulful nourishment to buffer emerging urbanisation. Olmsted’s approach to the design of urban greenspace as the embodiment of medicine conferred an instrumental value to urban nature that continues today.

Still, large-scale recreational nature-viewing, while more common than a century ago, remains unattainable for many biophilic individuals. Segregation from nature suggests these groups may be foregoing many of the health benefits derived from first-person nature exposure, opportunities to reduce stress among them. With societal trends showing lower frequency and increasing withdrawal from natured environments, both urban and wild, forms other than live nature deserves investigation as potential stress-reducing stimuli to offset this outdoor deficit. In a society in which psychological stressors become ever more pervasive, and mechanisms for reducing stress such as socialisation, outdoor physical activity, and nature contact routinely fewer, the importance of assessing nature exposure in all its dimensions, facsimiles, and delivery modes is not immaterial.

We return to a discussion of our summary findings where viewing nature in park and paintings elicited different GSR response patterns indicating dissimilar stress levels. Park viewers began with a lower rate of physiological arousal than the gallery viewers and a stable rate of relaxation. Confounding sensory input related to gallery viewing expectations, incongruence of finding seats in a normally open room, and a selection of a preferred painting likely occurred. Following that initial arousal, viewers exhibited lower response rates after the settling-in period, signalling deeper relaxation across the seven-minute viewing window.

Interesting conclusions can be drawn from comparison of readings related to the calming event, where participants were exposed either to nature paintings in the gallery or viewing of live nature in the park. In the case of the gallery, the levels of skin conductivity observed for study participants were at average levels. However, the readings were significantly lower when exposed to park viewing, which shows that the calming effect of park was much more pronounced than the corresponding effect of the landscape paintings in the gallery. A sunny, temperate 70°F day outdoors compared to an artificially lighted indoor gallery may have contributed immediately calming environmental conditions.

Indeed, the level of calming observed with STAI and POMS self-reported measures of anxiety and stress indicates that there is no significant difference between park and paintings viewing. As for the second finding, there is certainly a calming effect of each of the exposures in the study (comparing the beginning of the exposure with its later stages). In order to explain this divergence between self-reported and physiological measures we must underline the dual nature of measures used in the study. Self-reported measures, such as STAI and POMS, capture participants' subjective perceptions of their emotional state, which can be influenced by factors like self-awareness and social desirability and thus reflect more in line our expectations regarding stressful of calming impact of an event. In contrast, physiological measures like skin conductivity provide an objective assessment of stress responses that participants may not consciously recognise.

In this case, the self-reported measures suggest that participants perceived both environments as equally calming, while the physiological data reveal differences in the pace and pattern of stress reduction. We would like to emphasise that we cannot directly compare these two findings as they assess average level of perceived stress (STAI, POMS) or tendency in the evolution of stress levels. These findings also highlight the importance of using multi-method approaches to capture the complexity of stress responses, as different tools may tap into distinct aspects of these constructs.

We would like to stress that the higher initial arousal in gallery viewers may reflect contextual factors, such as the shorter walk to the gallery or the need to orient oneself to a single painting. We have revised the manuscript to include a discussion of this point and its potential implications for interpreting physiological data. Additionally, we have emphasised the complementary nature of self-reported and physiological measures in understanding stress responses.

However, in our study, it seemed to take longer for the participants to settle into viewing nature in the gallery. Perhaps the gallery was a less familiar environment, even for a museum-going audience. Over time, both treatments showed a reversal of arousal response. This may well have been an anticipatory effect as people knew there was a specific time limit for each experience. However, the effect was earlier and more pronounced in the gallery. Two explanations may account for this mid-point increase in the gallery GSR: either boredom with a completely static environment, or, as was expressed by some study participants in a post-experiment talk-back, the initial overall viewing of the painting was followed by more stimulating scrutiny of details.

### Explanation of artistic stimuli

Art viewing is not static but recalls memories and elicits meaning-making [81]. Far from an objective process, the experience of art viewing within a museum invites the personality and demographics of the observer, features of the artwork, and characteristics of the presentational context in relation to visitor behaviour. The process of artwork, moreover, occurs in stages, across which an initial pre-classification, perceptual analysis, memory, and attention to formal aspects of the artwork, and cognitive mastering are integrated in the viewer’s understanding and affective response [81]. The characteristics of the viewing audience during this study may have positively predisposed participants to an early acceptance of the art experience as one both authentic and important, given their extant knowledge of the artistic and commercial value of the landscape paintings under consideration. An audience less museum-inclined without this prior exposure or knowledge of the landscape collection’s importance may not perceive this influence, although may be equal susceptible to the awe-inspiring qualities of the viewed nature paintings. The field of neuroaesthetics in the outdoors is more preliminary, meanwhile, and less cognitively understood [82].

We recognise that rapid development of digital technologies is transforming how we interact with the outside world, *e.g.* the digital twinning of actual forests and hiking trails, so that multisensorial simulations of trees and natured spaces may overtake or replace virtual reality as a promising nature alternative. Whether a positive emotional response to these advancements in digitised nature can evoke pro-health responses may finally owe to some primordial rejection of overly synthesised nature. Or the reverse may unfold if the background extinction of authentic nature contact – owing to factors ranging from urbanisation, fear, or enthrallment with meta-reality – with nature surrogates increasingly replacing time spent outdoors.

Cognitive processing of art occurs in stages, where a comprehensive digestive phase of the work gives way to selective processing of individual details which make up the whole. The original relaxation phase can be overridden by an active cognitive uptake phase to a positive level of stimulus-response. This type of response is called affect circumplex and is well documented among art viewers [83,84]. Chirico et al. [85] discuss the subliminal aspect of awe common to both nature and art, as both are characterised by self-transcendence, perceptual vastness and a desire for shared experience, among other central dimensions. Likewise, in their study, neither art-based nor nature-based formats elicited a higher experience of the sublime [85]. The shared or communal experience of viewing nature in either treatment arm – gallery and park – may even have influenced, positively or negatively, the arousal factor registered in skin conductance. Even unspoken social interaction with study participants or facilitators may contributed to stress outcomes.

Higher initial arousal among gallery viewers may also relate to mood-based mnemonic recall, where participants more readily recall materials based on a given mood experienced with first exposure are prompted with subsequent exposure [86]. Thus, participants were primed for positive affect through mood congruence related to earlier familiarity with painted landscapes. Moreover, the landscapes on display are canonical representations of scenes that resonate firmly with viewers in terms of basic cognitive processing [87]. These scenes associate with mental images of landscapes congruent with their expected, remembered forms and thus reinforce connections previously made between mood and subject matter.

### Future research directions

Further exploration of this cognitive phenomenon of the nature experience through art is warranted. Future research directions might test synthetic nature, specifically paintings and holograms even, among audiences expanded beyond older museumgoers. These audiences should include participants non-habituated to viewing art and those working or living in institutional settings, such as break areas in hospitals and schools. A greater age range of more diverse participants would lend higher validity to these preliminary results. Additional biometrics would improve understanding of physiological response to the two exposures. These could include biomonitoring of heart rate variability, eye tracking, and electroencephalograms. Future forms of nature surrogacy may one day allow individuals currently disconnected from nature to explore the health benefits associated with nature exposure, potentially extending its reach to those adverse to the outdoors as well as those physically unable to participate in nature.

### Strengths and limitations

This study offered several strengths to address some gaps in the state-of-the-research which uses a nature surrogate as emotional health buffer. To the best of our knowledge, it is the first to directly compare the effectiveness of live nature to its inanimate proxy under conditions of biophysical monitoring. Physiological measurements this study tool produced removed potential biases of subjective data collection alone. At the same time, multiple methods of measuring response, including use of two validated psychological scales and confirmatory mixed methods design involving post-experiment audience discussion, broadened reliance on a single set of study outcomes. The design of a randomised crossover study in which participants acted as their own control limited potential time-invariant confounding factors while increasing statistical power. Most critically, this study leveraged the expertise of the Museum’s curatorial, education, and visitor services departments to engage research participants in viewing original paintings displayed within its galleries and in viewing of live nature from the Museum’s outdoor terraces which overlook an Olmsted Park.

Study limitations primarily concern our small and homogeneous sample. More than double our actual study participants number pre-registered their attendance; anticipation of the larger audience, given the fixed number of sensors sets, led to several participants being turned away at the event site. As a result, the homogeneous composition of 37 older white museum-going adults as study participants limits the generalisability and impact of our outcome. Their self-selection as research participants who knowingly enjoy art viewing introduces selection bias into the study. We should consider this study, therefore, a pilot which aims to expand to more diverse audiences over time. Study design neglected to include a control group of no nature viewing to observe stress reduction without any nature mediator. Lastly, repeated filling out of the STAI-POMS questionnaires at the conclusion of each transition may have inured participants to momentary accuracy of response, leading to loss of distinction in filling out the later forms.

## CONCLUSIONS

Natural landscapes and landscape paintings differed in their stress-reducing effects on participants in terms of physiological arousal but not on psychological calming. Our results indicate that transcendent landscape paintings may serve as a surrogate to time in nature, especially where live nature immersion may not always be suitable for physically and emotionally challenged individuals, those in institutional settings, or those generally disinclined toward spending time in nature. Given these circumstances, findings from this research could be relevant for populations without access to nature or averse to the outdoors yet could benefit from the stress reduction and attention restoration which nature viewing delivers. We conclude that landscape painter Frederic Church approaches the appeal of landscape architect Frederick Olmsted on stress reduction and approximates the positive affect recovery his park designs intended.

## Additional material


Online Supplementary Document
