# Supplementary material for: Stress reduction from landscape painting and live nature viewing: a comparative experimental study
Source: J Glob Health. 2025 May 16;15:04146. doi: 10.7189/jogh.15.04146 (PMC12082256; doi:10.7189/jogh.15.04146)
Supplement: Online Supplementary Document [file jogh-15-04146-s001.pdf]

Supplement to: Tomasso LP, Bialowolski P, Spengler JD. Stress reduction from landscape painting and live nature viewing: a comparative experimental study. J Glob Health. 2025;15:04146.

Figure S1

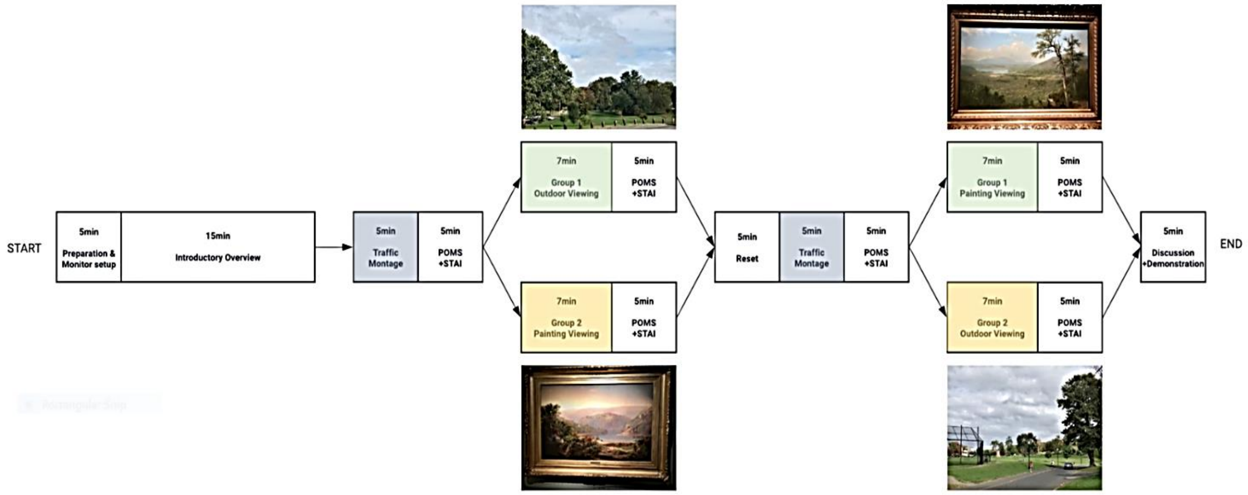

Figure S2

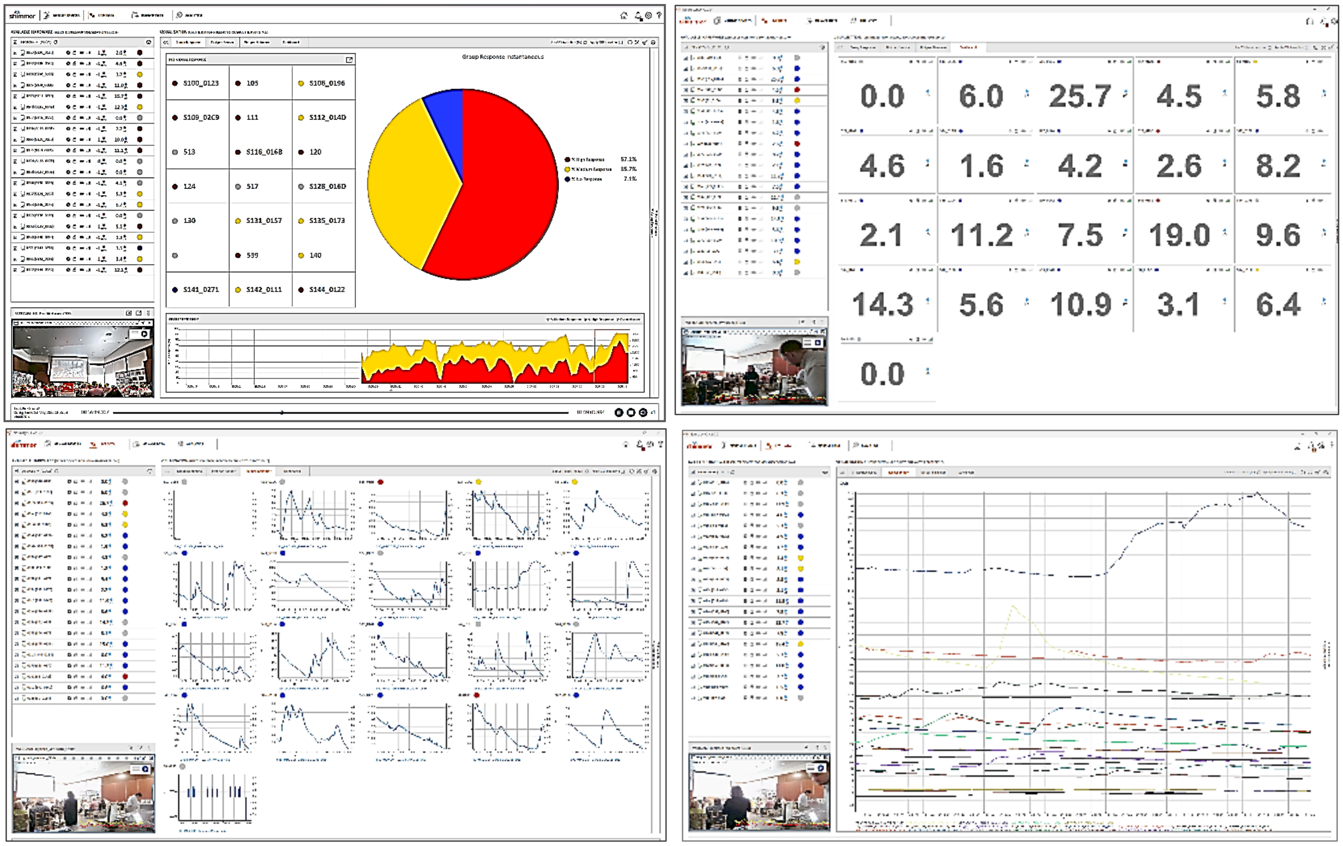

Table S1

**Overall**	**mean(SD) or # (%)**	**Overall**	**mean(SD) or # (%)**
**Number of Participants**	37	**Mental health** (%)	
**Age** (mean (SD))	68.51 (13.61)	excellent	14 (37.8)
**Sex** = (%)		very good	18 (48.6)
female	15 (67.6)	good	5 (13.5)
male	12 (32.4)	**Stress level** (mean)	2.68 (1.06)
**Race** = white (%)	37 (100.0)	**Rested** = yes (%)	32 (86.5)
**Childhood Urban Density** (%)		**Caffeine** = yes (%)	16 (43.2)
large city	4 (10.8)	**Nature affinity** (mean)†	5.03 (1.26)
suburb	23 (62.2)	**Frequency in nature** (%)	
small town	7 (18.9)	1-2x month	1 (2.7)
rural	2 (5.4)	1-2x week	8 (21.6)
mixed	1 (2.7)	3-4x week	13 (35.1)
**Physical health** (%)		daily	13 (35.1)
excellent	13 (35.1)	rarely	2 (5.4)
very good	19 (51.4)	**Positive nature experience** (%)	35 (94.6)
good	4 (10.8)	**Negative nature experience** (%)	2 (5.4)
fair	1 (2.7)		
